# Detection of Hydraulic Oil-Polluted Soil Using a Low-Cost Electronic Nose with Sample Heating

**DOI:** 10.3390/s26041154

**Published:** 2026-02-11

**Authors:** Piotr Borowik, Przemysław Pluta, Rafał Tarakowski, Tomasz Oszako

**Affiliations:** 1 Institute of Theory of Electrical Engineering, Measurement and Information Systems, Faculty of Electrical Engineering, Warsaw University of Technology, ul. Koszykowa 75, 00-662 Warszawa, Poland; 2 Forestry Students’ Scientific Association, Forest Department, Warsaw University of Life Sciences, Nowoursynowska 166, 02-787 Warsaw, Poland; s211238@sggw.edu.pl; 3 Faculty of Physics, Warsaw University of Technology, ul. Koszykowa 75, 00-662 Warszawa, Poland; rafal.tarakowski@pw.edu.pl; 4 Forest Protection Department, Forest Research Institute, ul. Braci Leśnej 3, 05-090 Sękocin Stary, Poland; t.oszako@ibles.waw.pl

**Keywords:** gas sensor, soil pollution, soil degradation, electronic nose, biodegradable oil, mineral oil

## Abstract

Monitoring soil contamination from petroleum products is vital for protecting human health and the environment. In forestry, hydraulic oil spills frequently result from leaks in equipment such as harvesters. This study evaluates a custom-built, inexpensive electronic nose, equipped with a Figaro TGS gas sensor array, for discriminating between pristine and contaminated soil samples. Two oil types and three pollution intensities were analyzed. The constructed electronic nose applied two sensor operation modes: (i) response to change of sensor operation condition from clean air to target odors and (ii) response to sensor heater temperature modulation. Classification was performed using Random Forest and Support Vector Machine (SVM) algorithms, and Linear Discriminant Analysis (LDA) was used to explore multidimensional data patterns. The sensor heater temperature modulation mode provided superior classification performance. Measurements at room temperature achieved an accuracy of 97%, clearly outperforming measurements on samples heated to 60 °C (75%). While the system successfully identified biodegradable oil contamination, standard mineral oil was more challenging to detect. Among the sensors tested, TGS 2602 was the most effective. These findings indicate that portable electronic noses can provide a statistically robust and cost-effective tool for assessing the severity of soil pollution.

## 1. Introduction

Soil quality monitoring is vital for environmental health and sustainable land management. Persistent pollutants like petroleum hydrocarbons and pesticides pose significant, long-term risks to ecosystems and human health. While conventional analytical techniques, such as gas chromatography–mass spectrometry (GC-MS), offer highly precise and sensitive results, they are typically expensive, time-intensive, and require dedicated laboratory facilities.

Electronic noses (e-noses) have recently emerged as a viable, rapid, and economical alternative for identifying soil pollutants. These multisensor devices emulate the human sense of smell to detect volatile organic compounds (VOCs) that are indicative of specific chemical or biological activity within the soil. A growing body of research confirms their effectiveness in identifying and differentiating between contaminants like hydrocarbons and pesticides [1,2,3], and even in assessing broader soil health indicators such as moisture content [4] or the presence of humic substances [5,6]. Although the first usage of an electronic nose to find soil contaminations was reported in 2010 by Rincón et al. [7], according to a bibliometric analysis made by Vidigal et al. [8] of the years 2012–2022, from all documents related to electronic noses, only 2% were about fuel-related applications.

Electronic nose (e-nose) technology has been implemented in diverse ways, spanning sophisticated custom-built sensor arrays, like those developed by Kong et al. [2], or the novel configurations proposed by Ruiz Gonzalez et al. [9]). E-noses can be used to distinguish between different types of oil contamination across various soil compositions, which helps evaluate the potential for reusing contaminated sites has been also investigated [10].

Additionally, Bieganowski et al. [11] demonstrated the feasibility of using an e-nose to assess soil pollution from gasoline and diesel across ten different soil matrices, specifically by analyzing how sensor signals change over time. Zaytsev et al. [12] advanced this capability by focusing on encoding the unique “smell patterns” of crude oil, thus enabling the precise identification of various specific contamination sources.

In forest environments, where access to traditional laboratories is often restricted, the use of affordable and portable monitoring solutions is essential. The category of low-cost electronic noses has emerged as an active field of research [13]. One example of this is a platform priced under 50 USD, designed for monitoring soil gas emissions, including those emanating from diesel-contaminated soil [14]. In previous studies [15], research of similar subject has been reported.

Similar sensors were used by Luan et al. [16] to find soil contamination by hydrocarbons. The laboratory analyses showed that it is possible to detect 1% of contamination in prepared soil. Although there are some limitations, it offers possibilities for the development of a device that can help to make decisions on soil recultivation.

When simple, low-cost devices are proposed, they are usually based on commercially available metal oxide (MOX) sensors. The electronic nose device [17], which was built for present research, utilized TGS (Taguchi Gas Sensors) and was designed to be a versatile tool. These gas sensors have proven effective at detecting volatile organic compounds across a wide range of applications, including environmental monitoring, industrial safety, or food quality control.

Another, current direction of development of electronic noses comprises the construction of our own sensors targeted to specific applications [18,19,20].

This study aims to investigate whether an inexpensive electronic nose can effectively differentiate between soil samples contaminated with standard hydraulic oil versus those polluted by the biodegradable alternative, both of which are frequently utilized in forestry machinery like harvesters. In managed forest landscapes, where hydraulic systems are widely used, even moderate spills may exceed legal thresholds for C_12_–C_35_ hydrocarbons, calling for simple on-site screening methods. An experiment was performed to verify the possibility of improvement of recognition accuracy using measurements by the electronic nose of heated samples. This leads to more effective evaporation from the samples but also changes the proportions of chemical components due to different evaporation temperatures of the ingredients. As Burlachenko et al. [21] emphasized, temperature is one of the most important parameters affecting the efficiency of VOC extraction in headspace methods. The idea that increased temperature may lead to the emission of additional chemical components that may be detected by an electronic nose was used by Bao et al. [22] to diagnose overheating of electrical components. Differences in the odor of milk at various temperatures were studied using an electonic nose [23]. Additionally, temperature effects registered by an electronic nose for classifying beef were studied [24,25]. The effect of temperature on the aroma release of yeast proteins was also studied [26]. Another goal of the research was the comparison of two modes of electronic nose operation, when both the data were collected during the adsorption phase of the sensor response and as a response to a change in TGS sensor heater temperature.

## 2. Materials and Methods

### 2.1. Preparation of Soil Samples

The soil used in this study was collected from a mature Scots pine (*Pinus sylvestris* L.) stand approximately 70 years old, located on a fresh mixed coniferous forest site. This soil type was selected because it is representative of typical Scots pine stands in Central Europe where hydraulic harvesters are commonly used and accidental oil spills may occur. In such stands, accidental hydraulic oil spills are a realistic risk. The soil itself is predominantly sandy, with low to moderate organic matter content, which favors relatively fast infiltration of oil and is frequently encountered on sites where pine plantations are established. For this reason, we considered this soil type an appropriate starting point for a proof-of-concept study, while recognizing that future work should extend the approach to other textures and organic matter contents.

Before sampling, the litter layer was carefully removed, and soil was excavated with a shovel from a maximum depth of 10 cm. Prior to the measurements, the soil was naturally air-dried for several days under laboratory conditions, allowing spontaneous moisture loss without the application of elevated temperatures or forced drying methods. This ensured moisture removal while preserving the soil’s inherent physicochemical properties, particularly those influencing VOC adsorption and release. No sieving was performed, meaning the material retained naturally occurring fragments of dead organic matter. The soil was predominantly sandy. During transport, it was stored in sealed plastic bags.

In the laboratory, the bulk soil was transferred into plastic containers and weighed so that each container held exactly 1 kg of material. Subsequently, the soil was contaminated with either hydraulic oil, Shell Tellus S2 VX46, (Shell plc, London, UK) or biodegradable hydraulic oil, Komatsu HE Gen II Natura (Kamatsu Ltd., Tokyo, Japan), both commonly used in forest harvesters. Contamination was applied using a syringe to allow precise dosing. Five variants of 1-kg samples were prepared.

- Control (uncontaminated): 0 mg/kg;
- Low mineral oil contamination: 300 mg/kg;
- High mineral oil contamination: 600 mg/kg;
- Low bio-oil contamination: 300 mg/kg;
- High bio-oil contamination: 600 mg/kg.

The lower level (300 mg/kg) corresponded to the maximum permissible concentration of the sum of C12–C35 hydrocarbons for category III land (forests) according to the Regulation of the Minister of the Environment of 1 September 2016 [27]. The higher concentration (600 mg/kg), twice the legal limit, was selected for experimental purposes.

Throughout the experiment, all five soil variants remained tightly sealed in plastic containers to prevent cross-contamination of odors. At the beginning of each of the five measurement days, smaller analytical samples were prepared. Soil was sub-sampled from the 1-kg containers into glass Petri dishes (80 mm diameter) and weighed to obtain 50 g per sample. These daily samples also remained closed except during electronic nose measurements. For each variant, three replicates were prepared, resulting in a total of 15 samples per day.

In this proof-of-concept study, we deliberately restricted the experiment to a single, predominantly sandy soil from a Scots pine stand, keeping moisture close to air-dry conditions, in order to reduce the number of varying factors and focus on the comparison between contamination types, contamination levels and e-nose operation modes.

### 2.2. Measurement Setup

#### 2.2.1. Electronic Nose Device

The experiment used the same device as in our previous works [15]. It consists of a set of the TGS series of gas sensor arrays manufactured by Figaro Engineering Inc. (Osaka, Japan), namely: TGS 2600, TGS 2602, TGS 2603, TGS 2610, TGS 2611, TGS 2612, and TGS 2620. The technical details of the electronic nose construction were described in previous papers, the gas chamber and mechanical part [17], and electronic circuit [28].

The applied TGS series sensors were designed with the principle to respond to the changes of gas presence [29]. Inside a sensor, the sensing material, typically tin dioxide, is heated by the built-in electric heater, usually made of platinum, up to the temperature of a few hundred Celsius degrees. However, gas detection and recognition may be also performed basing of the analysis of sensor response to the change of sensor heater temperature; these sensors can also be used for such tasks [30,31]. The choice of the sensor heater temperature modulation profile was studied in previous works [28,32,33,34].

Two types of sensor response can be collected by the used device: (i) the temporary value of the voltage representing sensor conductance over time, as a response to a change of conditions from clean air to the presence of the measured gas, and (ii) the voltage representing sensor resistance caused by a change in sensor heater voltage.

#### 2.2.2. Samples Heating Setup

To heat the samples for the subsequent measurements, a small circular heating plate (120 mm diameter) made of ABS plastic and tempered glass was used. The device was powered from the mains, consumed 18 W, and reached a temperature of 60 °C. However, direct heating of glass Petri dishes on the plate occasionally caused them to crack. This was likely due to uneven heat distribution and/or pre-existing microfractures in the glass.

**Figure 1:**
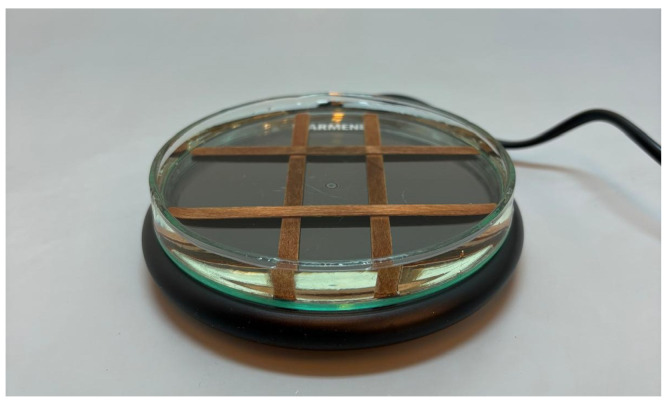
Improvised water bath setup used for heating soil samples.

To mitigate the problem of Petri dishes cracking during direct contact with the heating plate, an improvised indirect-heating system based on a water bath was constructed. The setup consisted of a larger glass Petri dish (110 mm diameter) partially filled with tap water and equipped with a mechanical support structure made of four wooden sticks. The sticks were arranged orthogonally in two perpendicular layers, forming a stable, cross platform at the bottom of the dish. This platform elevated the smaller sample-containing Petri dishes above the water surface, enabling uniform heat transfer through the water medium and eliminating direct exposure to the heating plate. The modified system effectively prevented thermal stress and cracking of the glassware during heating (Figure 1).

Before the heated measurements, the entire heating setup was pre-heated for 24 min: 12 min for the water bath alone and an additional 12 min with a test sample of uncontaminated soil. This was sufficient enough to obtain stable levels of temperature of the sample and the release of volatiles.

### 2.3. Measurements Using the Electronic Nose

Electronic nose measurements were conducted over five consecutive days, resulting in a total of 150 measurements—half at room temperature and half after sample heating—performed on 75 samples. The order of measurements each day was randomized using the Excel RAND function. Heated samples measurements followed the same randomized sequence as the room temperature measurements.

#### 2.3.1. Measurements at Room Temperature

**Figure 2:**
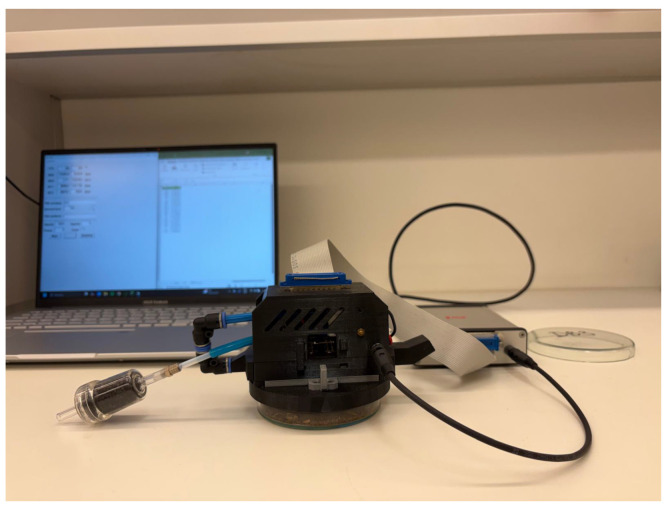
Measurement of a soil sample using the electronic nose.

At the start of each measurement day, the prepared samples were kept sealed in glass Petri dishes and were only opened at the moment of analysis. A single measurement sequence consisted of 1100 individual sensor readings (Figure 2).

The measurement sequence proceeded as follows. Initially, the sensor array was exposed to clean air to establish and stabilize the baseline signal (50 readings). After stabilization, the chamber was opened, allowing volatile compounds released from the soil sample to enter and interact with the sensors, thereby capturing their adsorption response (650 readings). Following this interaction phase, the chamber was closed again, allowing the sensors to recover and recondition themselves for the subsequent measurement (400 readings). Prior to measuring the actual samples, two preliminary test runs were conducted to verify the stability and proper functioning of the system.

#### 2.3.2. Measurements of Heated Samples

After completing all room temperature measurements, the samples were heated individually in the water bath system according to the previously established randomized order. Each sample underwent a 12 min heating period, during which it remained tightly sealed to prevent loss or mixing of volatile compounds. Immediately after heating, the still-closed sample was transferred to the measurement station. Upon opening the dish, the electronic nose measurement was initiated without delay and followed the same sequence as used for the room temperature measurements. Before commencing the full series of heated measurements, one test measurement was carried out on a heated sample to verify system stability.

We would like to admit that in our initial experimental design, we considered analyzing soil sample sensor responses across various temperatures. This approach could provide more detailed information, facilitating better differentiation between the studied samples. It assumes that contaminants consist of chemical components with distinct evaporation temperatures; thus, identifying an optimal sampling temperature could could potentially enhance accuracy of detection and differentiation. However, a significant obstacle to this approach is the long measurement cycle of the electronic nose. A single cycle exceeds 10 min, primarily due to the necessary sensor cleaning process. Consequently, collecting a representative number of observations, even at only two temperatures, requires several days of manual operations during measurements.

#### 2.3.3. Measurement Cycle

**Figure 3:**
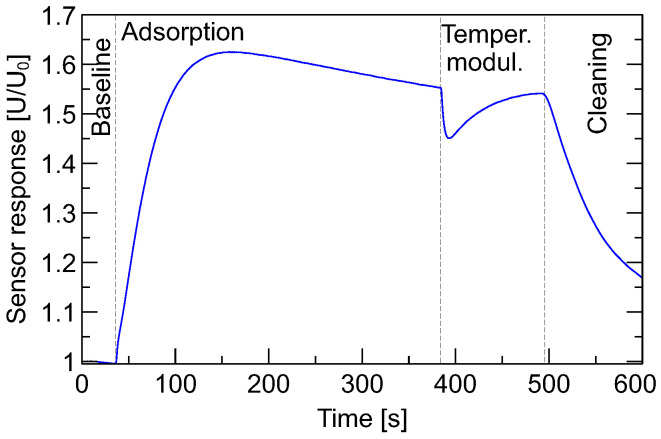
Example of a gas sensor response shape with indicated various phases of the masurements cycle.

In Figure 3, a typical shape of sensor response during a measurement cycle is presented. At the beginning of the measurement, sensors should be cleaned and free from the remains of gases from the previous measurement cycle.

At the beginning of the measurement, a baseline sensor response, when sensors are exposed to clean air conditions, is collected over a duration of 35 s, with readings every 0.7 s. After that, during the first phase of the measurement cycle, sensors are exposed to the measured gas, which lasts 385 s. During this so-called adsorption phase, the sensors’ heaters are supplied at a nominal, recommended by the manufacturer voltage of 5 V. In the following phase of the measurement cycle (indicated in the manuscript as temperature modulation), the sensor heater voltage drops to the level of 4.7 V, and this level is maintained for 105 s. The following sensor cleaning phase lasts 15 min. During that time, the sensor heater voltage is raised to its nominal voltage, and sensors relax in such conditions. The data were collected during the cleaning phase only for visualization purposes, but they were not used in the analysis.

### 2.4. Data Analysis

#### 2.4.1. Features Describing Gas Sensors Response

At the beginning, over a short period, the sensor response in clean air is collected. This baseline response is used for the first step of data preprocessing. According to the gas sensor manufacturer [29] recommendation, the sensor response relative to its response in clean air should be used. Thus, the measured voltage at each time moment (*U*), which is proportional to sensor conductivity, is transformed as $U/U_{0}$, where $U_{0}$ is the baseline magnitude.

As described in Section 2.3.3, the measurement data points are collected every 0.7 s and thus several hundred data points are collected from each sensor during a measurement cycle. Such data are not suitable for classical machine learning due to the extremely high dimensionality of the problem. The common procedure is to extract from the sensor response curves a hand-crafted set of features describing their shape [35].

In the presented analysis, the following features were extracted from the adsorption phase of the response curve: (i) area under the curve, which is equivalent to average sensor response, (ii) response level at the end of observation, (iii) maximum of the response, (iv) slope of the response at the beginning of the observation, (v) slope of the response at the end of the observation.

From the temperature modulation phase of the measurement cycle, the following features were extracted: (vi) area under the curve until minimum response is reached, (vii) area under the curve after minimum to the end of observation, (viii) total area under the curve, (ix) level of response at the end of observation, (x) curve slope ad the beginning of observation, (xi) minimum of the response.

Such features were extracted from each sensor response and then used as predictors for training the machine learning models.

Example of distribution of a sensor response feature for two temperatures of samples is demonstrated in Appendix A.

#### 2.4.2. Machine Learning-Based Classification

Electronic nose measurements use the collected data for the task of training machine learning classification models capable of discriminating between the classes under study. For this task, we used a Random Forest model of machine learning [36].

Random Forest is one of the most popular machine learning algorithms used for classification tasks. It uses a number of decision tree classifiers, trained on various sub-samples of the dataset and/or sub-samples of modeling features. Such an ensemble model uses the averaging of the results of the individual classifiers for a final prediction. Such an approach allows for fitting the model to nonlinear patterns in data with control of over-fitting. An important reason for the choice of this method was the fact that the algorithm embed variable selection method is robust regarding correlated features; such features are commonly found in electronic nose data.

For comparison purposes, we also used another popular classification algorithm—Support Vector Machine [37]—based on different theoretical principles. This was used to verify that the findings are supported by different classes of classification methods.

Python 3.12 language with the scikit-learn package [38] was used for data processing and machine learning modeling.

## 3. Results and Discussion

### 3.1. Accuracy of Classification

**Figure 4:**
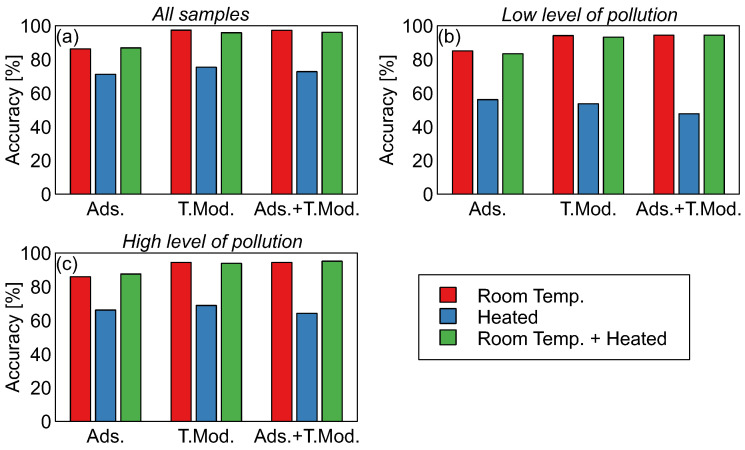
Accuracy of classification between the three considered sample categories: control, biodegradable, and mineral oil pollution. Results of the Random Forests model. Models trained with sets of modeling features extracted from various phases of sensor response, as indicated in the x-axis captions (adsorption, sensor heater temperature change, and both phases). Features extracted from sensor response in various measurement conditions, as indicated by bar color, are explained in the legend. Subfigures present data when modeling was performed using various sets of observations, as indicated in the charts: (**a**) all samples, (**b**) low level of pollution samples, (**c**) high level of pollution samples. Numerical results are presented in Table 1.

In Figure 4 and Table 1, a comparison of the results of the performance of machine learning models used for classification between the three categories of samples considered in this study (control, pollution by biodegradable, and pollution by mineral oil) is presented.

The accuracy of models was estimated using 10-fold cross-validation. Since the collected number of observations is limited, to avoid errors due to statistical fluctuations that may occur due to random splitting of the dataset to cross-validation folds, the procedure was repeated 20 times with different seeds of the random number generator, and such results were used for estimations of mean accuracy and its standard deviation. It was verified that a further increase in the number of repetitions does not change the presented results.

The obtained results of the model’s performance allow analysis of the data in several dimensions, based on the selection of modeling features and collected samples used for modeling.

#### 3.1.1. Temperature of Measured Samples

One of the main goals of the experiment was verification of the hypothesis that measurements of heated soil samples could be beneficial for the ability to differentiate between them using electronic nose measurement. At higher temperatures, more volatiles are released from the sample, but one can also expect that the proportion of chemical components constituting the odor of the sample changes. Other chemical components may vaporize due to differences in their evaporation temperature.

The results presented in Figure 4a and Table 1 demonstrate that measurements at room temperature had a classification accuracy ranging from 86.2% to 97.3%. Conversely, measurements after sample heating led to a classification accuracy of 71.1–75.3%. Also, the fusion of features collected by measurements of the same sample at two temperatures did not enable the obtainment of a significant improvement in the accuracy of classification.

This counterintuitive result merits reporting and may be informative for other researchers. It could be expected that sample heating could lead to a more abundant release of volatiles [21], which could have been helpful for detection and classification using devices applying gas sensors. Our measurements have not supported such an assumption. Similar conclusions were presented by Laga et al. [25] for differentiation between meat samples.

At this stage, we can only offer a plausible interpretation based on the known behavior of MOX sensors and the physical chemistry of VOC emission, rather than a definitive mechanistic explanation. First, heating the soil to 60 °C substantially increases the release of water vapour, which is known to strongly affect TGS-type sensors and can partially mask or distort responses to organic compounds. Second, at higher temperatures, the relative proportions of individual VOCs in the headspace may change, with more volatile components dominating the odor pattern and potentially reducing the contribution of lower-volatility compounds that carry important discriminatory information between contamination types. Third, the sensor responses themselves may become less linear or more saturated under these enriched and more humid headspace conditions, which could reduce the effective dynamic range available for discrimination.

#### 3.1.2. Phase of Measurement Cycle

Another goal of the analysis of the experimental data was the comparison of the usability of two modes of operation of the electronic nose and the performance of models trained on data collected during the adsorption phase of the sensor response and the models trained on data collected during the sensor heater temperature modulation phase.

As one can notice in Figure 4a and Table 1, in the case of samples measured at room temperature, the classification accuracy of the model trained using adsorption data reached 86.2%, while in the case where data from the heater temperature modulation phase were used, the accuracy of classification increased to 97.3%. This result demonstrates the advantage of applying sensor heater temperature modulation as a means to improve the performance of the electronic nose.

In the case of measurements of heated samples, both types of sensor response led to very similar classification accuracy, but as mentioned in the previous section, this was much lower than in the case of the measurements at room temperature.

The fusion of data collected during both types of sensor response did not improve classification performance. However, it should be noted that for such a high level of classification accuracy, any improvement is hard to achieve, and this result is not discouraging and may be different for other types of odors [32].

In our opinion, there is no direct correlation between two aspects of electronic nose measurements: sample heating and sensor heater temperature modulation. The operating temperature of the sensors is maintained by an internal heater; thus, the sensing element’s temperature should remain stable regardless of the sample gas temperature, even during temperature modulation. Given that the active element operates at several hundred degrees Celsius and the volume of gas entering the sensor chamber is very small, we believe that variations in sample temperature have a negligible impact on the results.

In the conducted experiment, utilizing two measurement methods with commercial gas sensors expanded the scope of data collection. This approach served to verify whether the results related to sample temperature modification remained consistent across both methodologies. Furthermore, comparing the results from these two methods and exploring the potential to fuse data from two independent measurements was an independent objective of this study.

#### 3.1.3. Severity of Soil Pollution

The data collected in the experiment consisted of polluted soil samples with two levels of pollution severity. In the results presented above, data from all measurements were used for model training and testing. It may be interesting to note what impact the severity of pollution has on the ability to differentiate between sample categories. In Figure 4b,c, the performance of two sets of models is presented. These models were trained and tested using subsets of collected data, either measurements of only control and low or control and high levels of pollution samples, respectively.

It can be seen that the results of the model’s accuracy obtained in the three considered cases are very similar. This is an encouraging result signifying that differentiation between unpolluted and polluted soil, by two types of hydraulic oil, can be achieved, even for low levels of pollution. Standard deviation of classification accuracy increased for the case of models trained on single levels of soil pollution datasets, which is related to higher uncertainty, when the number of training observations decreased.

#### 3.1.4. Choice of a Machine Learning Modeling Technique

The comparison and discussion of the results of machine learning models’ performance was based on the results of Random Forest models. For further verification of the found patterns in data, we performed the same types of analysis using another class algorithm, the Support Vector Machine. In both cases, very similar results were obtained, and the same patterns could be observed.

### 3.2. Importance of Gas Sensors

**Figure 5:**
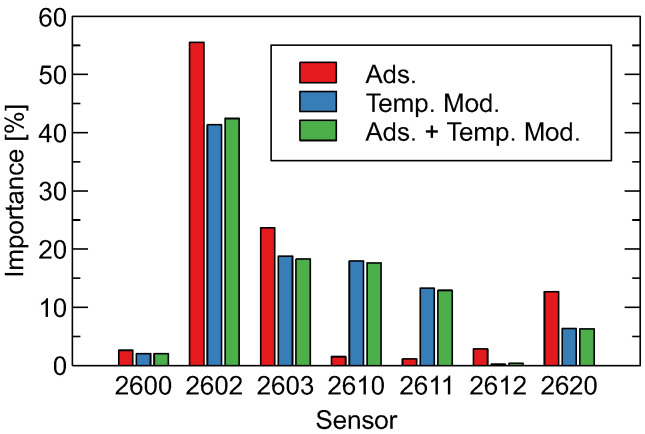
Importance of gas sensors for classification between the three considered sample categories. Results obtained using the Random Forests model, trained with observations collected at room temperature. Comparison of models using features extracted from various phases of the sensor response, as indicated by bar color.

In the case when a Random Forest classification model is applied, there is a possibility to extract from the model being trained a measure of the importance of features. The implemented method of Gini importance and mean decrease in accuracy measures how much the impurity (or randomness) within a node of a decision tree decreases when a specific feature is used to split the data [38]. The feature importance measure sums to 100%, and it is possible to aggregate them to the sensor level, which may be used as an indicator of the importance of a gas sensor for classification of samples categories (Figure 5). In our opinion, analysis of the importance on the sensor level is more meaningful than analysis of the importance of individual features.

Figure 5 shows that the response of sensor TGS 2602 contributes most to the classification, followed by TGS 2603. What else can be observed is that, for the case of classification based on modeling features extracted from the sensor heater temperature modulation phase of the sensor response, more sensors are involved. In particular, data extracted from sensors TGS-2610 and TGS-2611 have low importance for classification using adsorption-based features, but for the sensor heater temperature modulation-based method, the importance of these sensors is very close to that of TGS-2603.

On the one hand, this observation indicates that in the electronic nose using only the adsorption phase of the sensor response, the sensor array may be significantly reduced, as several sensors have no important role in providing useful data for classification.

On the other hand, such an observation may explain the results discussed above. An array with more sensors providing useful information was able to collect data, which, when using this data, made the classification of sample categories possible. But, as seen in the results of the analysis, the sensor heater temperature modulation response needs to be explored.

A greater interpretation of the results of selection of the most important sensors, allowing the collection of the data necessary for differentiation between samples categories, would be possible if differences in the chemical composition of emitted volatiles were known and could be compared with the specification of used sensors. We were unable to obtain detailed chemical specifications of hydraulic oils from the publications of product manufacturers. Biodegradable hydraulic oils typically contain synthetic esters and different additive packages compared to mineral oils, which are expected to translate into distinct VOC patterns; however, a rigorous confirmation of this assumption requires dedicated chemical analyses that are beyond the scope of the present study. Such data could typically be generated through Gas Chromatography–Mass Spectrometry (GC-MS) analysis. While this remains a limitation of the current study, we believe our findings still provide valuable insights into applications of cheap custom-made laboratory equipment.

According to the manufacturer’s information, TGS 2602 exhibits high sensitivity to odorous gases and a broad range of VOCs (including various organic solvents), while TGS 2603 is designed for detecting air contaminants associated with food spoilage and general indoor air quality. Both devices therefore target complex VOC mixtures rather than single, highly specific analytes, which is consistent with the multicomponent headspace generated by oil-contaminated soils.

The fact that TGS 2602 and 2603 were identified as the most important sensors in our Random Forest analysis aligns with their broad VOC sensitivity and suggests that the discriminative information in our experiment is carried by composite odor patterns rather than by a single, narrowly defined gas. It is also plausible that the biodegradable oil, which typically contains synthetic esters and a different additive package than the mineral oil, produces a more pronounced or distinct VOC signature in the sensitivity range of these sensors, contributing to the higher classification accuracy for biodegradable contamination.

### 3.3. Linear Discriminant Analysis

In the above part of the discussion, we described the results of classification performance, primarily using the Random Forest method. However, it may be instructive to also use the Linear Discrimination Analysis technique, which allows us to find a linear combination of features that best separates the considered classes of data. This is a supervised method, which we applied to the whole dataset, so there is a big chance of the model overfitting. However, this allows us to visualize the patterns in data, which can give some intuitive understanding.

**Figure 6:**
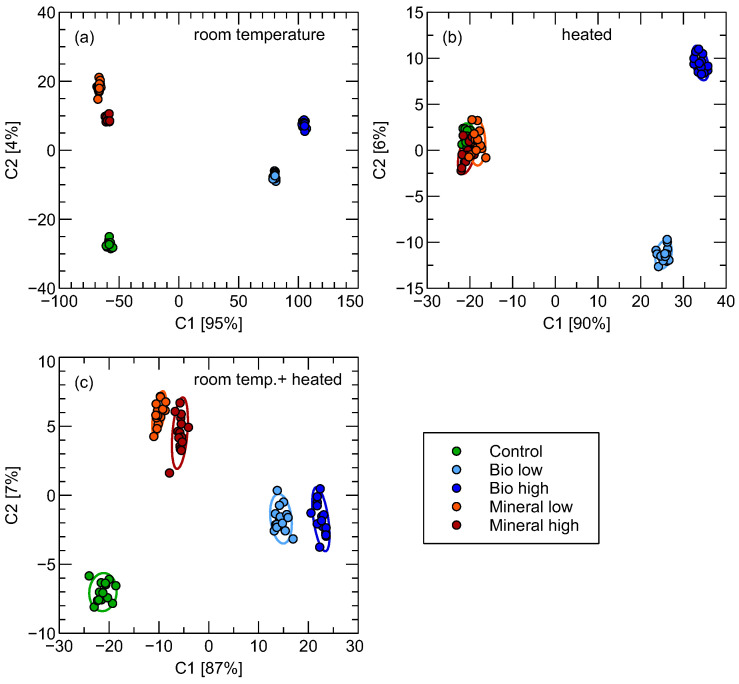
Visualization of the distribution of observations in the first two most important components directions of Linear Discrimination Analysis transformation. As input data, the features extracted from the two phases of the sensors’ response curves were used. The percentage of data variability captured by the components is indicated in axis labels. The subfigures present an analysis of the transformation of sensor response features collected in various soil sample temperatures, as indicated in the figures: (**a**) room temperature, (**b**) heated, (**c**) fusion of room temperature + heated.

As one can observe in Figure 6, room temperature measurements show distinctive clusters of data in the case of measurements of samples performed at room temperature. In the case of measurements of heated soil samples, a distinction between the control and samples polluted by mineral oil is not possible, at least using the first two the most important components.

## 4. Summary and Conclusions

A self-made, low-cost electronic nose applying TGS-type gas sensors manufactured by the Figaro corporation was used in the experiment. Two types of sensor data were collected: (i) time characteristics of sensor response to change conditions from clean air to the presence of the measured gas (gas adsorption), and (ii) dynamic sensor response to change of sensing material temperature, when the sensor was in the steady response state as immersed in the measured gas.

The device was applied to measurements of soil samples control (unpolluted) and polluted by two kinds of hydraulic oil—standard and biodegradable—in two levels of pollution severity. The measurement samples were performed at room temperature and then heated to about 60 °C. Random Forests and SVM classification models were used for differentiation between the samples, based on the measurement data.

Room temperature measurements allowed differentiation between sample categories with an accuracy of 86–97%. Heating of samples reduced discriminative power, and the obtained accuracy was ranged from 71 to 75%. The fusion of data from two temperatures measurements did not improve the accuracy of classification.

The advantage of measurements in the mode of sensor heater temperature modulation was observed with improvement of about 9% points of accuracy, when we compare the results based on sensor response to gas adsorption (86.2%) to sensor heater temperature modulation (97.3%).

It was confirmed that data collected by TGS 2602, followed by TGS 2603 sensors, play the most important role in differentiation between the studied categories of samples. It was also observed that in the case of measurements in temperature modulation mode, more sensors provide useful information, which explains the increase in the accuracy of classification.

A similar level of classification accuracy was obtained for models trained on data of low-level pollution, high-level pollution, and the whole collected dataset.

Our research suggests that measurements should be performed at room temperature and with sensor heater temperature modulation. The proposed electronic nose is a low-cost device, with components valued below 500 EUR [34]; adding heater voltage modulation remains cost-effective. Given that sample temperature significantly affects recognition performance, we recommend conducting measurements in a controlled environment rather than under field conditions.

## Figures and Tables

**Table 1:** Accuracy [%] of Random Forest (RF) and Support Vector Machine (SVM) machine learning models between the three considered sample categories: control, biodegradable, and mineral oil pollution. Mean of accuracy (Acc), and standard deviation (Std) calculated by k-fold cross-validation are presented. Models were trained using various sets of modeling features (i) extracted from two phases of sensor response (gas adsorption and sensor heater temperature modulation), presented in rows, and (ii) collected in various measurement conditions of soil sample temperature, presented in columns. Sub-tables represent results obtained for different sets of observations used for training, as indicated above.

Classification Using All Samples												
	**Room Temp. Sample**				**Heated Sample**				**Room + Heated**			
	**RF**		**SVM**		**RF**		**SVM**		**RF**		**SVM**	
	**Acc**	**Std**	**Acc**	**Std**	**Acc**	**Std**	**Acc**	**Std**	**Acc**	**Std**	**Acc**	**Std**
**Ads.**	86.2	11.7	87.5	10.6	71.1	12.7	67.2	14.2	86.8	10.1	83.6	12.0
**Temp. Mod.**	97.3	5.6	93.9	7.8	75.3	10.3	70.6	13.3	95.8	7.5	97.2	6.0
**Ads. + Temp.Mod.**	97.2	6.2	93.5	8.4	72.7	12.0	63.3	15.3	96.0	7.4	94.8	7.3
Classification for Low Level of Pollution												
	**Room Temp. Sample**				**Heated Sample**				**Room + Heated**			
	**RF**		**SVM**		**RF**		**SVM**		**RF**		**SVM**	
	**Acc**	**Std**	**Acc**	**Std**	**Acc**	**Std**	**Acc**	**Std**	**Acc**	**Std**	**Acc**	**Std**
**Ads.**	85.0	15.9	82.3	17.4	56.1	20.1	59.3	21.5	83.3	15.8	71.8	19.5
**Temp. Mod.**	94.1	10.9	89.5	12.6	53.6	19.8	60.4	18.7	93.2	12.4	97.7	6.7
**Ads. + Temp.Mod.**	94.3	10.3	93.6	10.8	47.7	19.8	55.4	22.5	94.4	10.8	93.5	10.9
Classification for High Level of Pollution												
	**Room Temp. Sample**				**Heated Sample**				**Room + Heated**			
	**RF**		**SVM**		**RF**		**SVM**		**RF**		**SVM**	
	**Acc**	**Std**	**Acc**	**Std**	**Acc**	**Std**	**Acc**	**Std**	**Acc**	**Std**	**Acc**	**Std**
**Ads.**	85.9	16.2	87.1	15.8	66.1	19.0	68.8	20.0	87.5	14.8	79.4	20.6
**Temp. Mod.**	94.4	11.1	95.3	9.1	68.8	20.2	80.4	15.2	93.8	11.6	92.0	11.1
**Ads. + Temp.Mod.**	94.4	11.0	89.9	12.3	64.2	19.6	71.6	20.3	95.1	9.4	92.0	11.7

**Figure A1:**
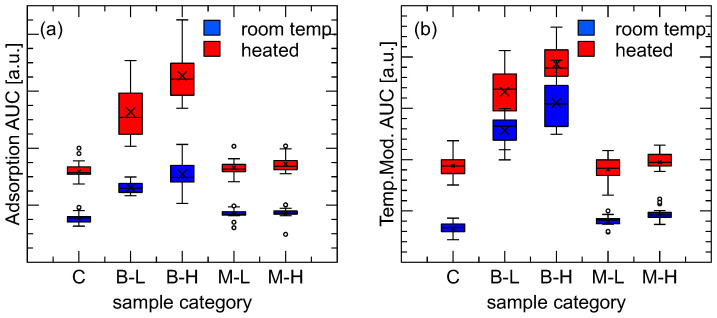
The feature “area under the curve” of (**a**) adsorption and (**b**) sensor heater modulation phase of the sensor response. The first character in the sample categories in the x-axis indicates sample type: C—control, B—biological oil, M—mineral oil. The second character indicates the level of soil pollution: L—low, H—high. Temperature of the sample is indicated by bar color.

## Data Availability

The original contributions presented in the study are included in the article. Further inquiries can be directed to the authors. The raw data supporting the conclusions of this article will be made available by the authors on request.

## Appendix A. Example of Sensor Response Feature

It may be interesting to present an example of a feature extracted from the sensor response curve (Figure 3) to demonstrate how it differs for various categories of samples.

In our opinion, a representative example may be area under the curve (Figure A1), which is extracted for both types of considered sensor response: adsorption and sensor heater temperature modulation. Additionally, it is worth noting that this feature is one of the most often used classification features extracted from the electronic nose data. In some studies, it is calculated as the average of sensor response during the observation period, but both definitions are completely equivalent.
